# Massive and Lengthy Clonal Nosocomial Expansion of Mycobacterium abscessus subsp. *massiliense* among Patients Who Are Ventilator Dependent without Cystic Fibrosis

**DOI:** 10.1128/spectrum.04908-22

**Published:** 2023-06-14

**Authors:** Kosaku Komiya, Mitsunori Yoshida, Sonoe Uchida, Shuichi Takikawa, Mari Yamasue, Takashi Matsumoto, Yuta Morishige, Akio Aono, Kazufumi Hiramatsu, Yoshio Yamaoka, Akira Nishizono, Manabu Ato, Jun-ichi Kadota, Satoshi Mitarai

**Affiliations:** a Respiratory Medicine and Infectious Diseases, Oita University Faculty of Medicine, Yufu, Oita, Japan; b Research Center for GLOBAL and LOCAL Infectious Diseases, Oita University Faculty of Medicine, Yufu, Oita, Japan; c Department of Mycobacteriology, Leprosy Research Centre, National Institute of Infectious Diseases, Higashimurayama, Tokyo, Japan; d Internal Medicine, National Hospital Organization Nishi-Beppu Hospital, Beppu, Oita, Japan; e Respiratory Medicine, Bungoono City Hospital, Bungoono, Oita, Japan; f Department of Environmental and Preventive Medicine, Oita University Faculty of Medicine, Yufu, Oita, Japan; g Department of Mycobacterium Reference and Research, the Research Institute of Tuberculosis, Japan Anti-Tuberculosis Association, Kiyose, Tokyo, Japan; h Department of Microbiology, Oita University Faculty of Medicine, Oita, Japan; Quest Diagnostics

**Keywords:** *Mycobacterium abscessus* subsp. *massiliense*, nontuberculous mycobacteria, transmission, whole-genome sequencing, progressive neurodegenerative disease

## Abstract

Nontuberculous mycobacterial infections are generally believed to be independently acquired from the environment. Although person-to-person transmission of nontuberculous mycobacteria, especially Mycobacterium abscessus subsp. *massiliense*, is a serious concern among individuals with cystic fibrosis (CF), evidence of its spread among patients without CF has never been established. We unexpectedly found a number of M. abscessus subsp. *massiliense* cases among patients without CF in a hospital. This study aimed to define the mechanism of M. abscessus subsp. *massiliense* infection among patients who were ventilator dependent and without CF who had progressive neurodegenerative diseases in our long-term care wards from 2014 to 2018 during suspected nosocomial outbreaks. We conducted whole-genome sequencing of M. abscessus subsp. *massiliense* isolates from 52 patients and environmental samples. Potential opportunities for in-hospital transmission were analyzed using epidemiological data. M. abscessus subsp. *massiliense* was isolated from one air sample obtained near a patient without CF who was colonized with M. abscessus subsp. *massiliense* but not from other potential sources. Phylogenetic analysis of the strains from these patients and the environmental isolate revealed clonal expansion of near-identical M. abscessus subsp. *massiliense* isolates, with the isolates generally differing by fewer than 22 single nucleotide polymorphisms (SNPs). Approximately half of the isolates differed by fewer than nine SNPs, indicating interpatient transmission. Whole-genome sequencing revealed a potential nosocomial outbreak among patients who were ventilator dependent and without CF.

**IMPORTANCE** The isolation of M. abscessus subsp. *massiliense* from the air, but not from environmental fluid samples, may suggest airborne transmission. This was the first report to demonstrate person-to-person transmission of M. abscessus subsp. *massiliense*, even among patients without CF. M. abscessus subsp. *massiliense* may spread among patients who are ventilator dependent without CF through direct or indirect in-hospital transmission. The current infection control measures should address potential transmission among patients without CF, particularly in facilities that treat patients who are ventilator dependent and patients with preexisting chronic pulmonary diseases, such as CF.

## INTRODUCTION

Nontuberculous mycobacteria (NTM) are ubiquitous environmental organisms found in water and soil that cause subacute or chronic pulmonary infectious diseases in susceptible individuals, particularly those with preexisting lung diseases such as cystic fibrosis (CF) (1–4). Mycobacterium abscessus, including M. abscessus subsp. *abscessus*, M. abscessus subsp. *massiliense*, and M. abscessus subsp. *bolletii*, have emerged as significant respiratory pathogens, and their prevalence is significantly increasing in the United States and Asia-Pacific region (5–7). Approximately 10% of individuals with CF in the United States and Europe appear to be infected with M. abscessus (8, 9). These pathogens display intrinsic multidrug resistance to several antibiotic classes and inducible resistance to macrolides. It can therefore be difficult to treat M. abscessus infections sufficiently, even with prolonged combination antibiotic therapy (10). Treatment failure accelerates the decline in lung function and potentially leads to poor prognosis in patients with CF (4).

NTM infections are believed to be independently acquired from the environment (11). However, recent studies indicated that M. abscessus subsp. *massiliense* can be transmitted among patients with CF (12, 13). Aitken et al. reported an M. abscessus subsp. *massiliense* outbreak at a CF center in the United States involving five patients with CF (12). In the study, the genomic sequences of M. abscessus subsp. *massiliense* isolates from different patients were nearly identical and more closely related than strains repeatedly isolated from the same patients. In another study, Bryant et al. used whole-genome sequencing (WGS) to identify two clustered outbreaks involving 11 patients infected with identical M. abscessus subsp. *massiliense* isolates at a CF center in Papworth, United Kingdom (13). They found that the clinical isolates from different patients were almost genetically identical. These isolates shared the same mutations responsible for multidrug resistance. This, together with the fact that comprehensive environmental sampling failed to detect a potential NTM infection source, suggested that direct transmission is possible among patients with CF in the clinic. M. abscessus subsp. *massiliense* infection is a significant threat to individuals with preexisting lung diseases. The exact transmission route and mechanism must be clarified to protect these high-risk patients from infection.

Recent population-level genomic analyses illustrated that most M. abscessus infections are acquired through global dominant circulating clone (DCC) transmission, which is associated with worse clinical outcomes of CF and increased virulence in cell-based and mouse infection models (14–16). Most patients with CF are infected with clustered isolates, and strains isolated from patients with CF in outbreaks in the United Kingdom and United States were classified as DCC3 (14). Current transmission networks include individuals with and without CF, and it is difficult to speculate whether patients with CF, who are unlikely to travel globally, mainly spread M. abscessus globally. Hence, no solid evidence of transmission between healthy individuals and patients with CF has been established; however, it is possible that DCCs initially colonize populations without CF and then spread throughout the CF community. Thus, the transmission mechanism among both communities with and without CF requires clarification. However, no M. abscessus outbreaks among patients without CF have been reported.

We isolated M. abscessus subsp. *massiliense* from the respiratory secretions of 52 patients who were ventilator dependent and without CF with progressive neurodegenerative diseases in the long-term care wards of a single hospital in Japan in 2014 to 2018. Environmental samples from potential infection sources, including water and air in the wards, were collected. A single M. abscessus subsp. *massiliense* strain was successfully isolated from the air around a patient without CF colonized with M. abscessus subsp. *massiliense* but not from other potential sources. To determine the mechanism by which M. abscessus subsp. *massiliense* was transmitted among these patients, we assessed this possible nosocomial outbreak using WGS and epidemiological data.

## RESULTS

### Patient characteristics and the results of the environmental survey.

This study included 52 patients who were ventilator dependent with tracheostomy because of progressive neurogenerative diseases and had at least one positive culture for M. abscessus in lower respiratory samples obtained using a suction cannula through the tracheostomy orifice. The median age was 64 years, 50 patients (96%) required tube feeding, and the median duration of long-term care hospitalization exceeded 3,000 days (see Table S3 in the supplemental material). Twenty-nine patients had been hospitalized since 2013 or earlier, but no M. abscessus strain was isolated. The major reasons for sputum examination for acid-fast bacillus (AFB) culture were screening when NTM were isolated from another patient in the same ward, followed by evaluations of fever, respiratory symptoms, inflammatory markers, and abnormal chest radiographs. Sample examinations of AFB culture were repeated more than once in 42 of the 52 patients (81%), and positive culture results were confirmed more than once in 32 patients. The colony morphologies were documented in 37 cases as follows: smooth in 10, rough in 2, and a mixture of smooth and rough in 25 cases.

No patient was clinically suspected to have M. abscessus subsp. *massiliense* infection, and no patient received antibiotic therapy for NTM infection. Of the 52 patients, 11 (21%) died of other infectious diseases, cardiac failure, malignancy, or renal failure, but no deaths from M. abscessus subsp. *massiliense* infection were observed.

In the environmental survey, 38 specimens (all water and swab suspensions) were culture negative. Only one air sample collected from a patient’s room was culture positive as a mixture of smooth and rough colonies, and the Nishi-Beppu (NBP) isolate (NBP-5F41) was identified as M. abscessus subsp. *massiliense* by subsequent genomic analyses.

### Phylogeny of the clinical and environmental isolates.

**FIG 1:**
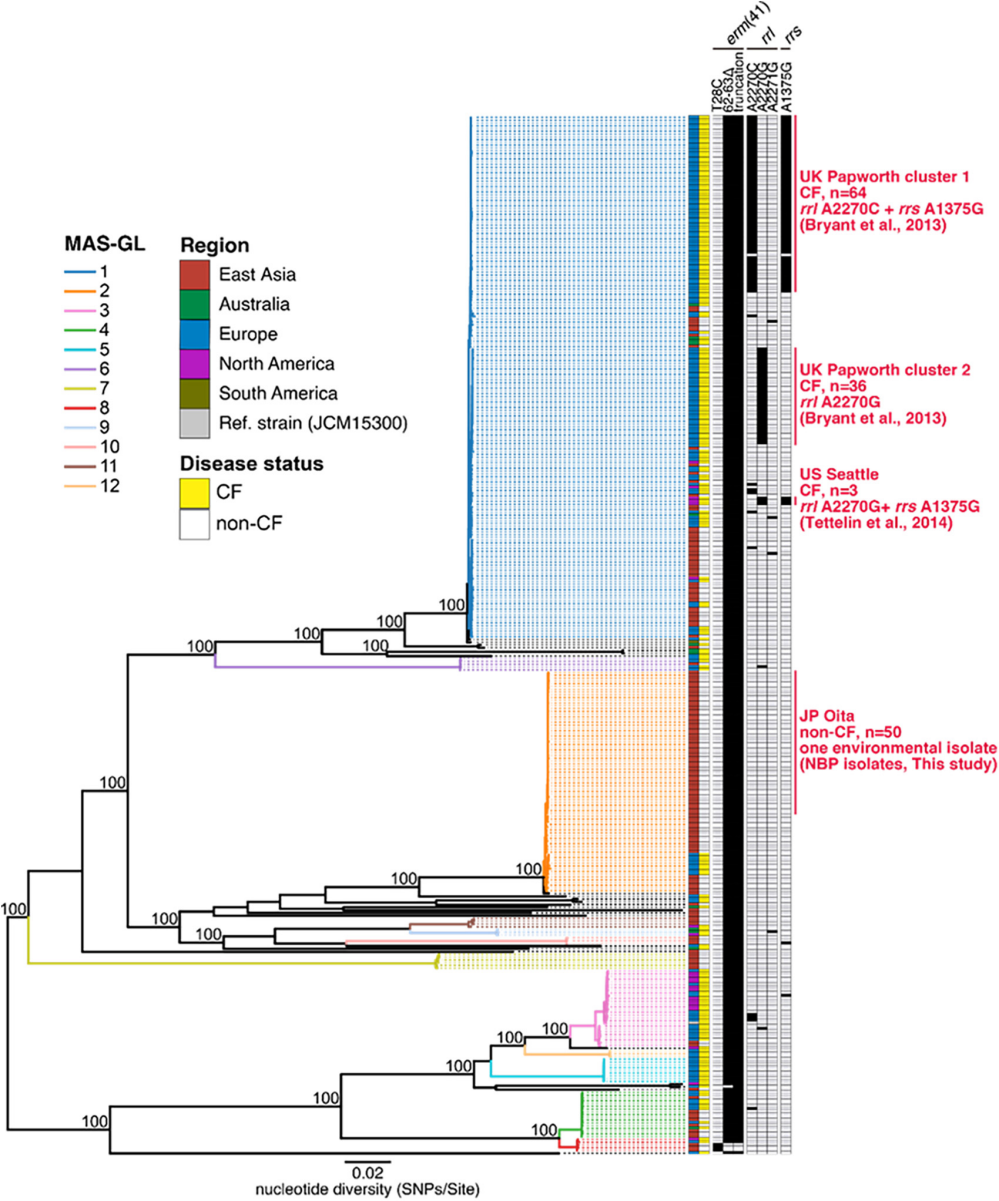
Phylogenetic association of isolates in the current study with other isolates in the global Mycobacterium abscessus subsp. *massiliense* population. A core genome alignment of 376 isolates from five regions (Australia, East Asia, Europe, North America, and South America) was used to determine the phylogeny of the global M. abscessus subsp. *massiliense* population, including NBP isolates. The complete genome sequence of JCM15300 was used as a reference. The recombination-free alignment in the core genome was used with RAxML to construct a maximum likelihood tree with 1,000 bootstrap replicates. The 12 monophyletic clusters (MAS-GL1 to MAS-GL12) identified using TreeGubbins are presented. Each color box corresponds to the region from which the clinical isolate was isolated. The corresponding patients’ disease statuses (cystic fibrosis [CF] in yellow and non-CF in white) are presented. The presence (black) and absence (white) of macrolide or aminoglycoside resistance-associated mutations are indicated. The scale bar indicates the mean number of nucleotide substitutions (SNPs) per site on the respective branch.

M. abscessus subsp. *massiliense* isolated during previous nosocomial outbreaks at CF hospitals was genetically related to DCC3 (15, 17). We previously found that DCC3 strains are distributed but not necessarily dominant among patients without CF in East Asia (18). Therefore, we first investigated whether the NBP isolates from our patients were genetically related to DCC3. The maximum likelihood tree indicated that all NBP isolates grouped in a phylogenetic cluster (MAS-GL2; *P* < 0.001) that was distinct from previously reported outbreak strains in CF hospitals in the United Kingdom and United States (Fig. 1). The MAS-GL2 cluster, including NBP isolates, was phylogenetically distinct from DCC3, but was the second-most-prevalent strain in patients without CF in Japan and Taiwan (18). We also found that NBP-5F41 clustered tightly with other NBP strains in MAS-GL2.

Because clinical isolates from previous nosocomial outbreaks in CF hospitals shared mutations associated with macrolide and/or aminoglycoside resistance, as presented in Fig. 1 (13, 16, 19), we next asked whether NBP isolates (including the environmental isolate) carried the same mutations. There were no point mutations of residues 2270 and 2271 of the 23S rRNA (*rrl*) gene or residue 1375 of 23S rRNA (*rrs*) in all NBP isolates.

### Detection of possible clonal expansions among global M. abscessus subsp. *massiliense* populations.

**FIG 2:**
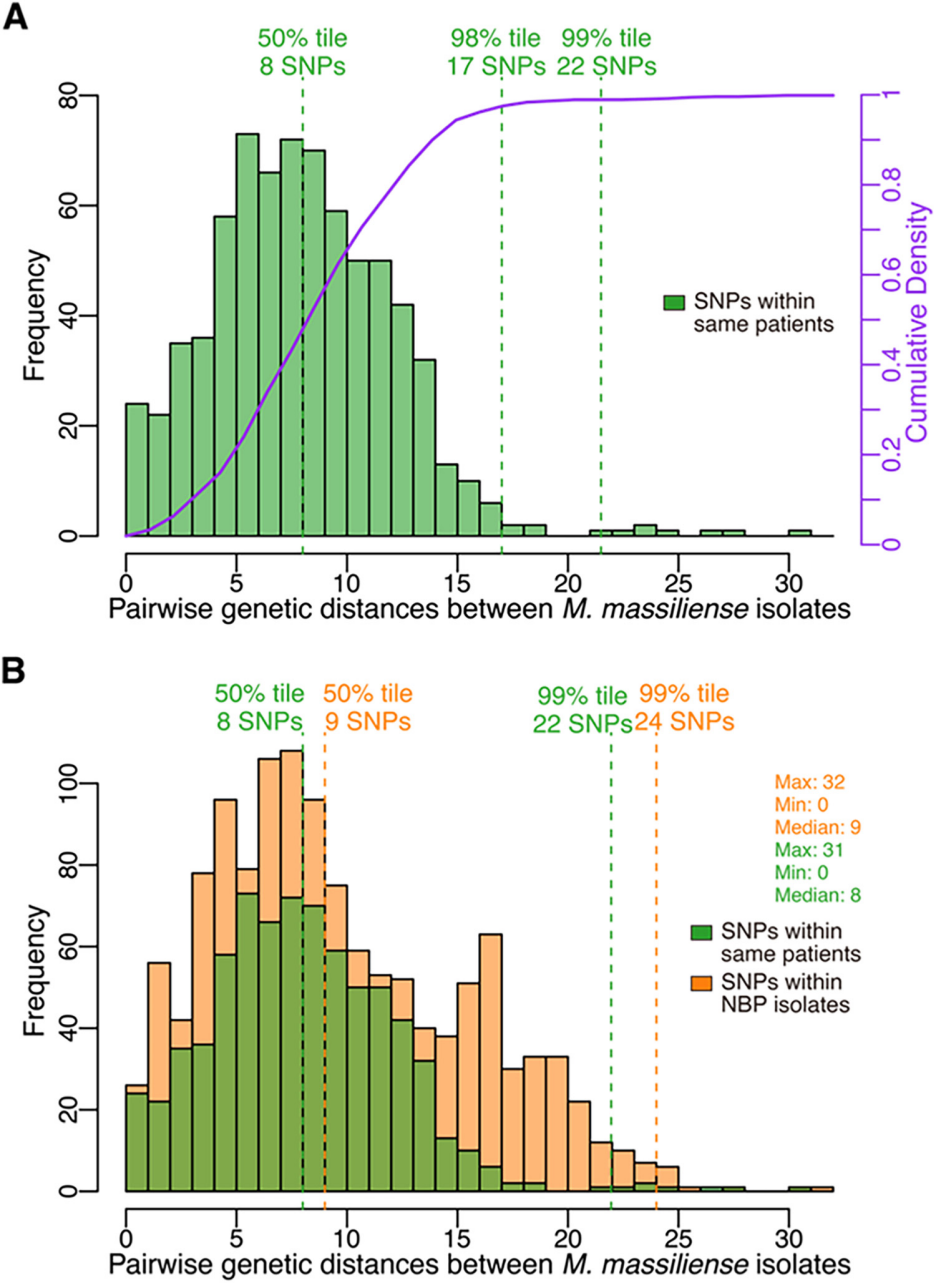
Distribution of pairwise genetic distances among Mycobacterium abscessus subsp. *massiliense* isolates. (A) Histogram of pairwise single nucleotide polymorphism (SNP) distances among M. abscessus subsp. *massiliense* isolates consecutively obtained from the same patients. As described in Fig. 1, the whole-genome alignment was used to calculate SNP distances among isolates using snp-dists (https://github.com/tseemann/snp-dists). The 99th, 98th, and 50th percentiles of the SNP distances among isolates are presented. The purple line indicates the cumulative density of these SNP distances. (B) Comparison of pairwise SNP distances within NBP strains and those within clinical isolates constitutively isolated from the same patients.

We analyzed the genetic distance between our M. abscessus subsp. *massiliense* isolates to assess whether different patients were infected with genetically identical bacteria. In our analysis pipeline, the maximum genetic distance in the global population of M. abscessus subsp. *massiliense* (*n* = 376) was 14,984, and the maximum genetic distance between isolates belonging to each MAS-GL cluster was 256 (see Fig. S1 in the supplemental material). In these phylogenetic clusters, the genetic distances between strains repeatedly isolated from the same patients were as much as 31, and more than 98% and 99% of isolates carried fewer than 17 and 22 single nucleotide polymorphisms (SNPs), respectively (Fig. 2A). The SNP distances among the NBP isolates were comparable to those within the same patients (Fig. 2B).

**FIG 3:**
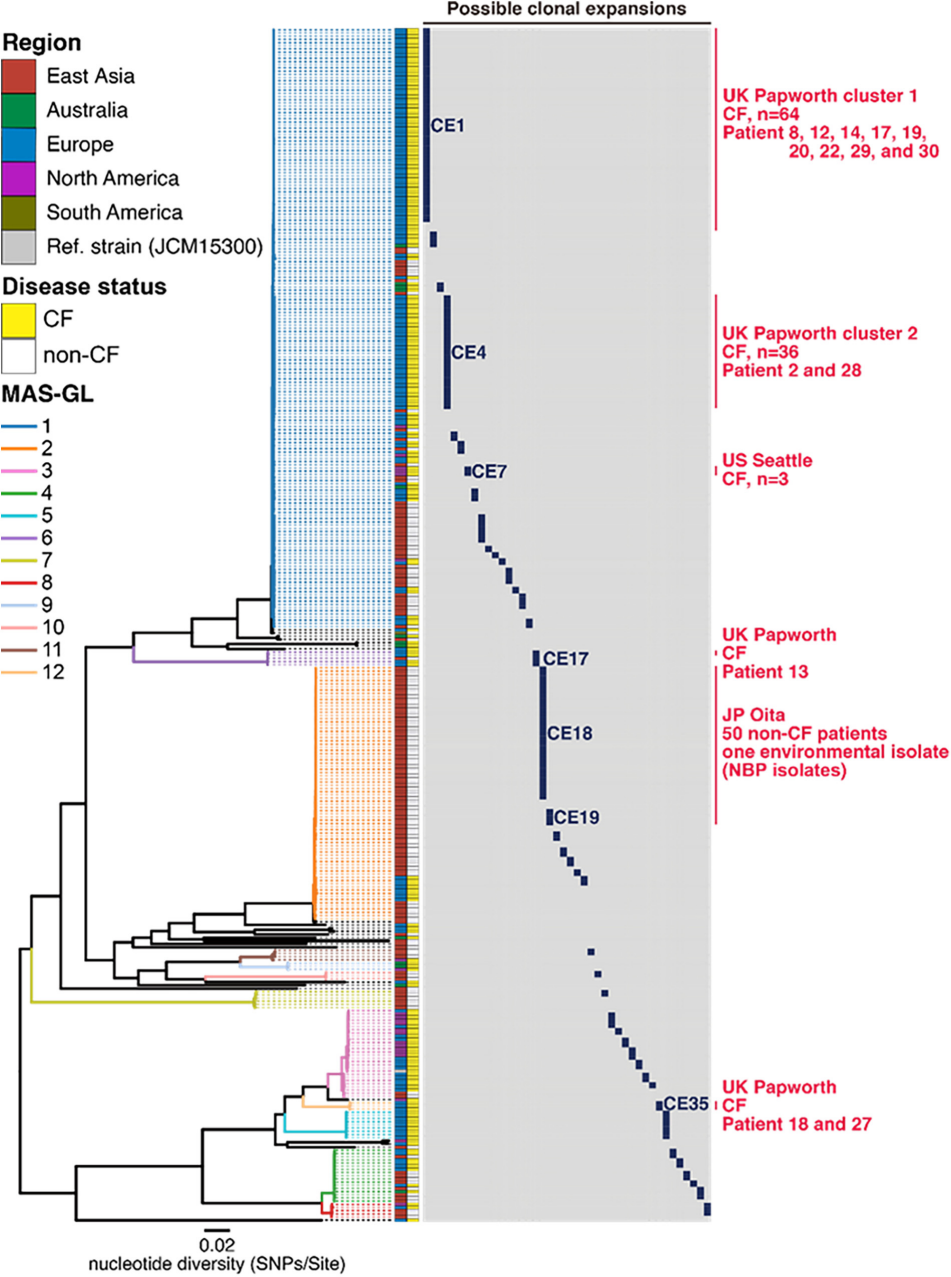
Possible clonal expansions among Mycobacterium abscessus subsp. *massiliense* isolates. rPinecone software was used to detect possible clonal expansions (CEs) in the M. abscessus subsp. *massiliense* population. The phylogenetic tree described in Fig. 1 was converted to a single nucleotide polymorphism (SNP)-scaled tree using pyjar. This resulting tree and the SNP threshold (20 SNPs, accounting for 98.5% of SNPs among M. abscessus subsp. *massiliense* isolates consecutively obtained from the same patients [Fig. 2]) were used as inputs for rPinecone. Blue boxes indicate detected CEs. The indicated phylogenetic tree was estimated as described in Fig. 1. The 12 MAS-GL clusters, the disease status (cystic fibrosis [CF] or non-CF) of the corresponding patients, and the region from which the clinical isolate was obtained are shown in Fig. 1.

Using a root-to-tip directional approach to define sublineages within a phylogenetic tree according to the SNP distance from the ancestral node (20), we detected 42 possible clonal expansions (CEs) in the M. abscessus subsp. *massiliense* population (SNP threshold = 20) (Fig. 3). All clinical isolates repeatedly isolated from the same patients with CF were identified as CE1, CE4, CE7, CE17, or CE35, excluding two isolates (14a and PAP728). In particular, CE1, CE4, and CE7 contained clinical isolates potentially acquired via human-to-human transmission (based on the clinical information of the patients collected during previous nosocomial outbreaks at CF hospitals). At an SNP threshold of 17 to 22, the clinical and environmental isolates of NBP appeared to represent an expansion of two clones, CE18 and CE19 (Fig. 3; Fig. S2).

### Genomic structures exclusively shared among the NBP clinical and environmental isolates.

**FIG 4:**
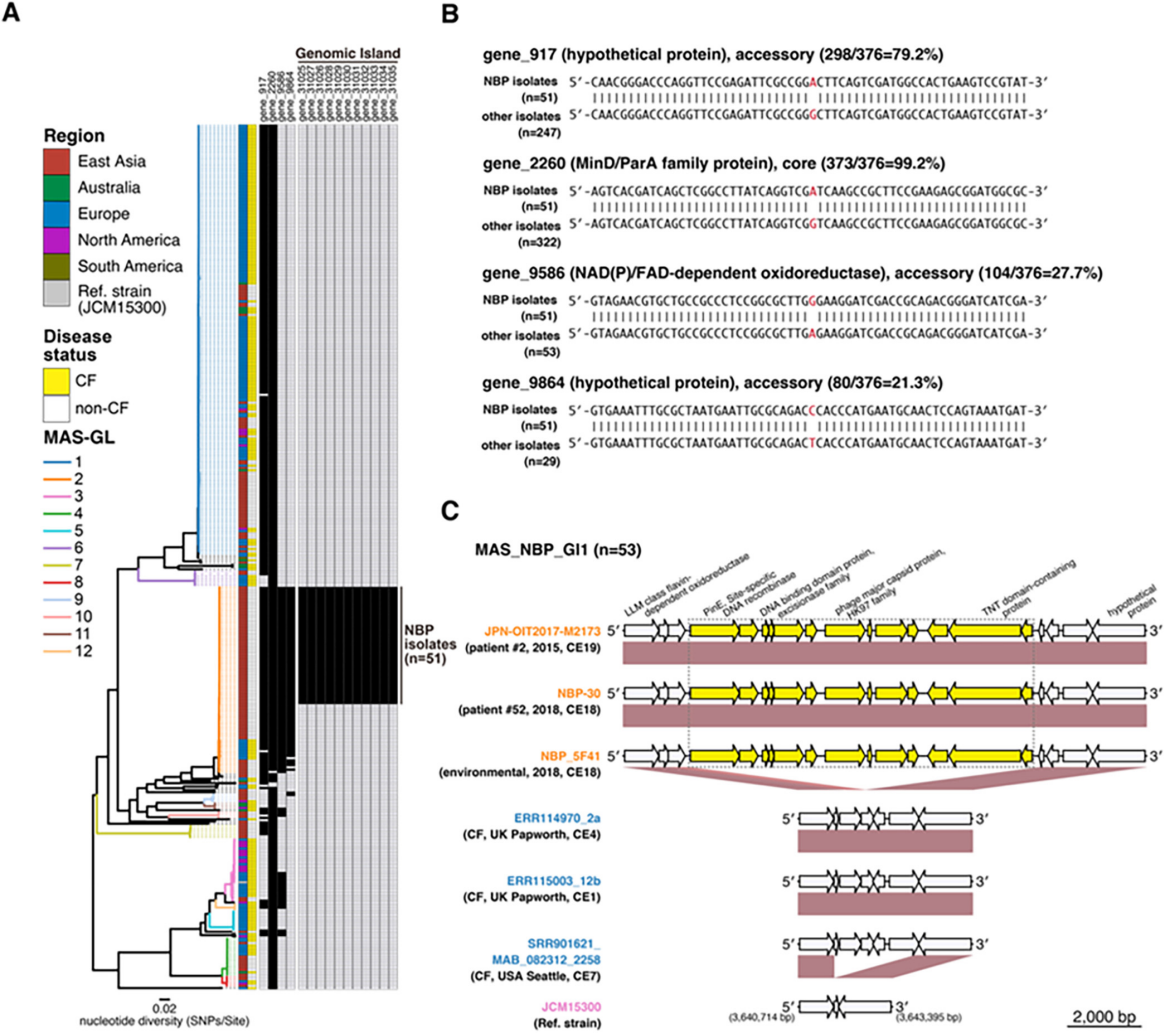
Genomic signatures shared among Mycobacterium abscessus subsp. *massiliense* NBP isolates. (A) The presence (black) and absence (white) of genes that distinguish NBP strains from other strains are indicated. Phylogenetic trees were determined as described in Fig. 1. The 12 MAS-GL clusters, the disease status (cystic fibrosis [CF] or non-CF) of the corresponding patients, and the region from which the clinical isolate was obtained are presented in Fig. 1. (B) Uniting pairs containing substitutions between NBP isolates (*n* = 51) and other M. abscessus subsp. *massiliense* strains; (C) genomic island exclusively shared among NBP clinical and environmental isolates. Arrows indicate genes annotated with DFAST-core (27). Orthologous genes between clinical isolates are presented in red. Thirteen genes exclusively associated with NBP clinical and environmental isolates are presented in yellow.

During previous nosocomial outbreaks at CF hospitals (13, 16), genetically similar strains were isolated from different patients. They shared characteristic mutations associated with macrolide and/or aminoglycoside resistance (Fig. 1). We examined the genomic features distinguishing NBP strains from other M. abscessus subsp. *massiliense* strains. Using a bacterial genome-wide association study method, we sought characteristic SNPs in mycobacterial genes shared exclusively among the NBP isolates. Consequently, we obtained reads for 23,102 genes in the M. abscessus subsp. *massiliense* population (Fig. S3), and the NBP strains exclusively shared characteristic SNPs in one core gene (gene_2260) and three accessory genes (gene_917, gene_9586, and gene_9864) (Fig. 4A and B). In addition, the NBP isolates had a genomic island encoding 13 genes (MAS_NBP_GI1, ~9,840 bp) that were not present in other M. abscessus subsp. *massiliense* strains (Fig. 4A and C).

### Clinicoepidemiological analysis.

**FIG 5:**
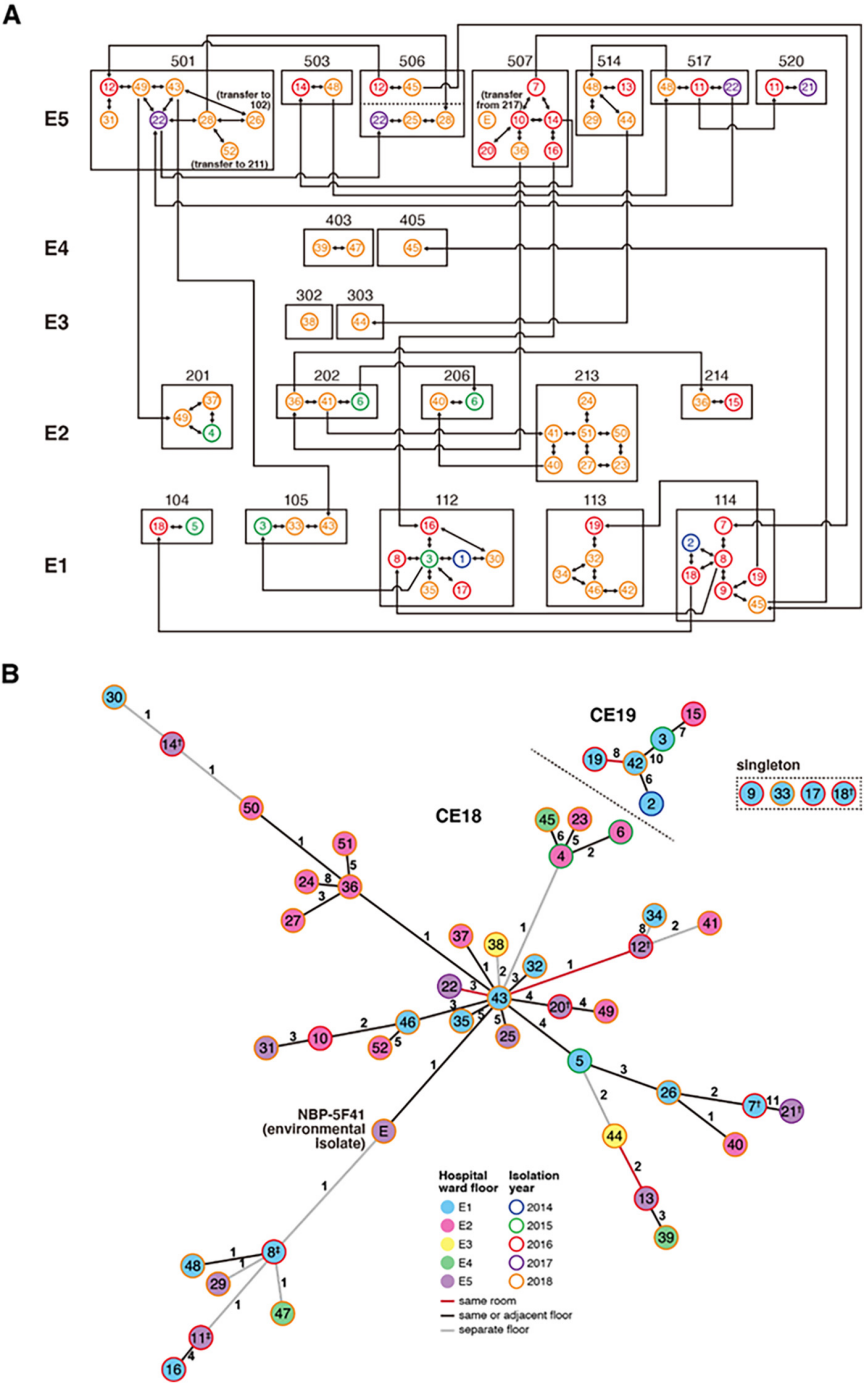
Opportunities for patient-to-patient transmission among NBP patients. (A) History of patient room transfers. A single-headed arrow denotes patients moving between rooms, and a double-headed arrow denotes patients in the same room at the same time. The number beside the double-headed arrow indicates the single nucleotide polymorphism distance. The timing of Mycobacterium abscessus subsp. *massiliense* isolation is displayed by different colors as follows: blue (patients 1 and 2) in 2014, green (from patients 3 to 6) in 2015, red (from patients 7 to 20) in 2016, purple (patients 21 and 22) in 2017, and orange (patients 23 to 52) in 2018. The dotted line for room 506 indicates contacts at different times. (B) Minimum-spanning network of M. abscessus subsp. *massiliense* NBP clinical and environmental isolates. Using the same alignment as in Fig. 1, a minimum-spanning network within CE18 and CE19 was constructed using the igraph R package. Each circle’s outer or inner color represents the year and ward in which the corresponding clinical or environmental isolate was isolated. The number in each circle represents the patient number (listed in Table S1) from which each clinical isolate was isolated. The red lines indicate that there are records of two corresponding patients admitted to the same room, the black lines indicate that there are records of two corresponding patients admitted to the same or adjacent floor, and the gray lines indicate that the corresponding patients were admitted to distant floors.

To infer the mode of transmission of M. abscessus subsp. *massiliense* within our hospital, clinical information from the 52 patients was analyzed in combination with a network analysis of the clinical and environmental NBP isolates. The 52 patients were housed across five wards on five separate floors within the same building (east ward floors E1 to E5). Based on the location of the patient rooms and the history of previous room transfers, it was confirmed that all patients, excluding patients 38, 39, and 47, had shared a room with an M. abscessus subsp. *massiliense*-positive patient. Rooms 302 (where patient 38 stayed) and 403 (where patients 39 and 47 stayed) were adjacent to rooms where M. abscessus subsp. *massiliense*-positive patients who were transferred from different floors stayed (Fig. 5A). In addition, we conducted a timeline analysis of potential patient-to-patient transmission. Figure 5A illustrates the first contact with other patients before the identification of M. abscessus subsp. *massiliense*; however, all patients but patient 38 had numerous opportunities of sharing a room with each other both before and after M. abscessus subsp. *massiliense* identification (Fig. S4). Therefore, the timing of M. abscessus subsp. *massiliense* identification does not denote initial infection because the reasons for sample examinations of AFB culture varied (e.g., screening or evaluation of symptoms).

A minimum-spanning network of NBP isolates illustrated that a few isolates were connected to many isolates. For instance, the isolates from patients 43 were connected to 13 isolates. In comparison, many others were connected to three or fewer isolates (Fig. 5B). Notably, the clinical isolate from patient 43 was closely related to the isolates from patients hospitalized on other floors. Some of these events can be explained by the records of patient room transfers (Fig. 5; Table S1). In 2018, M. abscessus subsp. *massiliense* was isolated from patient 43 (NBP-21) on floor E1 (room 105). This patient was previously admitted on floor E5 (room 501). NBP-21 was closely related to JPN_OIT2017_M2159 and NBP-07, which was isolated from patients 12 and 22, respectively, who were admitted to the same room as patient 43. NBP-11, derived from patient 25, was related to NBP-21, and patients 12, 22, and 25 were previously admitted to the same room (room 506). Furthermore, NBP-21 was found to be closely related to the isolate of patient 20, who was admitted on floor E5, and to the environmental isolate NBP-5F41, which was isolated in the room into which patient 20 was admitted. In addition, the isolate from patient 36, related to NBP-21, was isolated on floor E2. This patient was transferred from the same room as patient 20 on floor E5.

Similar cases were observed in addition to patient 43. The isolates from patients 44 and 13 (NBP-32 and JPN_OIT2017_M2167, respectively) were closely related. Although patient 44 was also staying on floor E3 when M. abscessus subsp. *massiliense* was isolated, this patient had previously been admitted to the same room as patient 13 on floor E5 (room 514). The isolate of patient 42 (NBP_19) was related to the isolates of patients 9 and 2. It was found that patients 42 and 19 were in the same room when M. abscessus subsp. *massiliense* was isolated, and patient 19 was transferred from the room in which patient 2 had stayed (Fig. 5; Table S1). In our environmental survey, we could not isolate M. abscessus subsp. *massiliense* from common facilities (e.g., water sources and ventilation outlets) in wards E1 to E5, and only one strain was isolated from air samples from a hospital room.

## DISCUSSION

We detected clonal expansion of near-identical M. abscessus subsp. *massiliense* strains among patients who were ventilator dependent and without CF but with progressive neurodegenerative diseases. These isolates differed by fewer than 22 SNPs, and almost half of the isolates varied by fewer than nine SNPs. This variation represented less diversity than observed within isolates from a single individual, as reported previously (13, 15, 21). Frequent patient transfers between hospital rooms provide numerous opportunities for contact with patients colonized with M. abscessus subsp. *massiliense*. In particular, isolates from bedridden patients staying on separate floors were nearly identical in several cases. Our environmental survey isolated M. abscessus subsp. *massiliense* from the air around an infected patient but not from other potential sources. This strain was genetically identical to strains derived from patients who stayed in the same room. Based on these observations, it is plausible that patients colonized with M. abscessus subsp. *massiliense* spread the bacteria by moving between hospital rooms rather than patients individually contracting M. abscessus subsp. *massiliense* from the environment.

A recent study performed WGS of NTM isolates collected from the Colorado Adult CF Program, and the findings revealed no genetic similarity between respiratory and health care environmental isolates (22). The authors suggested that health-care-associated transmission of M. abscessus is rare, whereas the presence of genetically similar isolates is insufficient to determine the transmission route. Admittedly, interpatient transmission cannot be proven by genetic similarity alone. However, we did not detect M. abscessus in any samples taken from environmental sources such as water or showerheads in the wards’ common facilities. This suggests that ventilator-dependent individuals were unlikely to independently acquire M. abscessus subsp. *massiliense* from the hospital environment. Patients with neurodegenerative diseases require intensive care, including frequent suctioning of airway secretions over a long period. Airway suctioning, mechanical ventilation, and oral care are aerosol-generating procedures (23). Thus, we presume that M. abscessus subsp. *massiliense* spreads via medical workers, equipment, or aerosols generated through health care techniques. The isolation of M. abscessus subsp. *massiliense* from an air sample taken from a room where an M. abscessus carrier stayed supports this hypothesis. Furthermore, three patients (38, 39, and 47) stayed in rooms adjacent to those occupied by carriers, further suggesting aerosol transmission.

Interestingly, no patients were treated for M. abscessus subsp. *massiliense* infections, even when M. abscessus isolates were continuously obtained. In patients who underwent computed tomography (CT), centrilobular nodules with cavities, which are characteristic of severe M. abscessus subsp. *massiliense* infection, were not observed, which likely explains the lack of treatment. Furthermore, no patients assessed in this study died of M. abscessus subsp. *massiliense* infection. Thus, unlike patients with CF, M. abscessus subsp. *massiliense* colonization does not appear to affect the prognosis of ventilator-dependent patients with progressive neurodegenerative diseases. However, some patients without CF with M. abscessus infection may have progressive disease; thus, M. abscessus subsp. *massiliense* infection has the potential to worsen prognosis even among patients without CF.

Although patients were not clinically suspected to have NTM infection when examined by an acid-fast bacillus sputum test, M. abscessus subsp. *massiliense* isolates were found in their respiratory secretions. Nishi-Beppu National Hospital has a tuberculosis ward, and physicians can quickly perform the acid-fast bacillus test in their laboratories, thereby identifying outbreaks of M. abscessus subsp. *massiliense* among patients without CF with progressive neurodegenerative diseases. Undiagnosed M. abscessus subsp. *massiliense* infection or colonization might exist, particularly among patients who were ventilator dependent and without CF requiring intensive respiratory care. Although M. abscessus subsp. *massiliense* might not affect the prognoses of patients without preexisting chronic pulmonary diseases, it can be amplified in their airways and then spread to high-risk patients (14). Furthermore, the maximum likelihood tree indicated that all NBP isolates that grouped in a phylogenetic cluster were distinct from previously reported outbreak strains in CF hospitals in the United Kingdom and United States. The environmental isolate identified in this study was also found to tightly cluster with other NBP strains in MAS-GL2, which is a significant phylogenetic cluster in Japan and Taiwan (18).

The current study indicates the possibility of direct person-to-person transmission of M. abscessus through aerosols among patients with non-CF for the first time, as M. abscessus subsp. *massiliense* was isolated from an air sample taken from the wards but not from water sources (24). Thus, the findings of the current study could help prevent the future spread of this pathogen to patients with preexisting chronic pulmonary diseases such as CF in health care settings.

However, this study had several limitations that warrant discussion. First, because this was a retrospective study, the timing of M. abscessus isolation and reasons for the mycobacterial culture order were not standardized. The study design may have resulted in biased patient selection, making it impossible to obtain accurate data regarding M. abscessus transmission among patients. Lack of sequence data for M. abscessus strains consecutively isolated from same patients also limited bioinformatic analyses, especially in the determination of SNP cutoffs. In addition, we were forced to use strains that are phylogenetically distant from the NBP isolates as reference genome sequences, which limited the amount of information available for our alignment-based SNP/phylogenetic analyses. To further improve the resolution of these analyses, it is necessary to determine the complete genome sequence of a more closely related strain and use it as a reference sequence for downstream analyses. Second, the environmental survey was conducted only once in 2017, after the identification of M. abscessus subsp. *massiliense* as being the same species based on a variable number tandem repeat (VNTR) analysis. We systematically screened M. abscessus in the potential infection sources in the wards; however, the timing was not the same for the majority of the isolations observed in 2018, which might not directly suggest airborne transmission (25). Other potential sources that this study did not test, such as ventilators placed on patients, might have been missed as an infection mechanism. Furthermore, independent acquisition from the environment cannot be ruled out based on the absence of detection of M. abscessus in the environment, as the presence of an environmental mycobacterium could be underestimated because of isolation difficulties. Third, it is still uncertain whether M. abscessus subsp. *massiliense* isolation denotes infection or colonization. Respiratory samples were obtained by suction cannula through tracheostomy orifice, which would not deny colonization on the tracheostomy tube. At least, no patient was clinically suspected to have M. abscessus subsp. *massiliense* infection, and no patient received antibiotic therapy for NTM infection. Finally, contact transmission by medical workers was not examined in the environmental survey. These limitations need to be addressed in the future to elucidate the mode of transmission of M. abscessus.

In conclusion, this is the first report demonstrating possible person-to-person transmission of M. abscessus subsp. *massiliense* through aerosols among patients without CF. M. abscessus subsp. *massiliense* isolates obtained from ventilator-dependent patients with progressive neurodegenerative diseases were genetically similar, and the strain isolated from a ward air sample was identical to the respiratory isolates. M. abscessus subsp. *massiliense* was not isolated from any other potential infection source, indicating few opportunities for independent acquisition from the static environment. However, M. abscessus detection in the environment may have been underestimated, and we have conducted the environmental survey only once. M. abscessus subsp. *massiliense* might spread among patients without CF through direct or indirect in-hospital transmission. Current infection control measures should address the potential for M. abscessus subsp. *massiliense* transmission among patients without CF, particularly in institutes that treat ventilator-dependent patients and patients with preexisting chronic pulmonary diseases such as CF. For effective infection control measures, a large-scale (e.g., nationwide) screening of patients who are ventilator dependent, as well as systematic environmental survey in the case of the confirmation of multiple positive cases, would be required to validate the results of this study.

## MATERIALS AND METHODS

### Patients and sample collection.

M. abscessus subsp. *massiliense* was isolated from respiratory samples collected from 52 patients with progressive neurodegenerative diseases during 2014 to 2018. We collected information on the patients’ backgrounds, reasons for the mycobacterial culture order, and the treatment administered after M. abscessus subsp. *massiliense* was isolated. The dates and locations of the patients’ beds, which were obtained from the medical information database, are listed in Table S1 and summarized in Table S2 in the supplemental material. We conducted a screening of all patients who were ventilator dependent with progressive neurogenerative diseases in 2018 exclusively. At that time, 100 patients were screened for AFB culture and M. abscessus subsp. *massiliense* was identified in 30 cases (30%).

We stored the clinical isolates as the frozen aliquots of mycobacterial growth indicator tube samples. We analyzed all recoverable isolates from each patient with M. abscessus, as detected by DNA-DNA hybridization. After initial culture on solid medium, we collected colony swabs for subculture. Antibiotic susceptibility testing was performed using BrothMIC NTM (Kyokuto Pharmaceuticals, Tokyo, Japan) based on Clinical and Laboratory Standards Institute document M24.

### Environmental survey.

In November 2017, after the identification of M. abscessus subsp. *massiliense* as being the same species by VNTR, we randomly collected 39 environmental specimens: 18 water samples, 12 fomite swabs, and 9 air samples. Tap water (50 mL) and swab specimens were collected from the patients’ rooms, bathrooms, and nursing stations, which were commonly used by the hospitalized patients. The water was centrifuged at 3,000 × *g* for 20 min at 4°C, and the sediment was treated with *N*-acetyl-l-cysteine (NALC)–NaOH according to a standard method. The swab specimen was resuspended in 5 mL of distilled saline, followed by the same pretreatment method. The pretreated specimens were inoculated into MGIT (Becton, Dickinson, Tokyo, Japan) and incubated for up to 6 weeks. Air sampling was performed in patients’ rooms and bathrooms using an SAS super IAQ instrument (VWR, Radnor, PA, USA), which collected 1,000 L in 10 min. The collected air was directly blown into Middlebrook 7H10 medium supplemented with 10% OADC (oleic acid-albumin-dextrose-catalase), 500 mg/L cycloheximide, and PANTA (polymyxin B, amphotericin B, nalidixic acid, trimethoprim, and azlocillin) (Becton Dickinson). The culture plates were incubated in a 5% CO_2_ incubator at 30°C for up to 8 weeks.

### WGS and genomic analyses.

A WGS library was constructed from the clinical and environmental isolates using the QIAseq FX DNA library kit (Qiagen, Hilden, Germany). Sequencing was performed on the standard MiSeq platform (Illumina Inc., CA, USA) using the MiSeq reagent kit v.3 (600 cycles) (Illumina, Inc.) with 350-mer × 250-mer paired-end short reads according to the manufacturer’s instructions.

### Statistical analysis.

WGS data for each isolate were quality filtered (minimum length of 25 and Phred quality score of 20) using the Sickle software (https://github.com/najoshi/sickle) and *de novo* assembled using the Shovill pipeline (https://github.com/tseemann/shovill) with the adapter trimming option. Assembly statistics are presented in Table S4. Two samples (NBP-10 and JPN-OIT2017-M2171) were excluded from the downstream genomic analyses because of a high proportion of nonself reads. Each isolate’s subspecies were determined using average nucleotide identity (ANI) values among M. abscessus clinical isolates as described previously (26). Further details on the methods used for genomic analyses are presented in Text S1 in the supplemental material.

### Data availability.

All raw read data of newly sequenced strains used in this study have been deposited into the DNA Data Bank of Japan and the National Centre for Biotechnology Information under BioProject accession no. PRJDB13427.
