# Supplementary material for: Massive and Lengthy Clonal Nosocomial Expansion of Mycobacterium abscessus subsp. massiliense among Patients Who Are Ventilator Dependent without Cystic Fibrosis
Source: Microbiol Spectr. 2023 Jun 14;11(4):e04908-22. doi: 10.1128/spectrum.04908-22 (PMC10433864; doi:10.1128/spectrum.04908-22)
Supplement: Supplemental file 1 — Supplemental material. Download spectrum.04908-22-s0001.pdf, PDF file, 1.0 MB [file spectrum.04908-22-s0001.pdf]

Supplemental material

Massive and lengthy clonal nosocomial expansion of *Mycobacterium abscessus* subsp. *massiliense* among patients who were ventilator dependent without cystic fibrosis

Kosaku Komiya MD, MPH, PhD, Mitsunori Yoshida PhD, Sonoe Uchida MD, Shuichi Takikawa MD, Mari Yamasue MD, PhD, Takashi Matsumoto PhD, Yuta Morishige PhD, Akio Aono, Kazufumi Hiramatsu MD, PhD, Yoshio Yamaoka MD, PhD, Akira Nishizono MD, PhD, Manabu Ato MD, PhD, Jun-ichi Kadota MD, PhD, Satoshi Mitarai MD, PhD

Supplemental Methods

Genomic analysis of Mycobacterium abscessus subsp. massiliense (M. massiliense) isolates

To analyze the phylogeny of *M. massiliense* isolates in this study, we combined our data set with publicly available whole-genome sequencing data from 330 *M. massiliense* clinical isolates, including clinical isolates consecutively isolated from patients involved in previous nosocomial outbreaks in the UK and USA (1-4). Additional raw read data were obtained from the Sequence Read Archive, subjected to quality filters (minimum length: 25, Phred quality score: 20) using the Sickle software, and assembled *de novo* using the Shovill pipeline With the "--trim" option. After assembly, the percentage of contaminating reads was estimated using the CheckM2 software (5), and six samples that contained more than 5% of nonself-reads were excluded. As described elsewhere (4, 6), to assess the quality of the assembly of each isolate, the genomic fraction of each isolate (percentage of alignment to the reference genome *M. massilisense* JCM15300) was calculated and was greater than 88% for all samples. Phylogeny estimation and single-nucleotide polymorphism (SNP) detection were conducted as described previously (4) with some modifications. In brief, we performed pairwise genome alignment between a complete genome sequence of *M. massiliense* JCM 15300 (Accession number; AP014547) and an isolate draft sequence using the MUMmer package (7). We then combined all of the alignments into a whole-genome alignment (3,827,236 bp, covering 76.8% of the reference genome), in which each position corresponded to that of JCM15300 using custom Perl scripts. The findings of Gubbins (8) were used to infer recombination sites. After masking 5,893 detected recombinogenic sites, this alignment was used to construct a maximum-likelihood tree using RAxML ver. 8.2.12.(9) The resulting phylogenetic tree was used to identify significant phylogenetic clusters ($P < 0.001$) in the *M. massiliense* population using TreeGubbins (3) as previously described (4). As previously mentioned, the phylogenetic tree and associated data were visualized using ggtree R package (10). Furthermore, a minimum spanning network of the Nishi–Beppu (NBP) clinical and environmental isolates was assessed using the igraph R package (11) and visualized using the ggplot2 package.

Possible clonal expansions in the *M. massiliense* population were explored as previously described with some modifications (6). The recombination-free whole-genome alignment was used to calculate the SNP distances among *M. massiliense* isolates using snp-dists (https://github.com/tseemann/snp-dists). We investigated the distribution of SNP distances between consecutive clinical isolates from the same patient. We then used rPinecone software to detect possible clonal expansions (12) using the SNP distance among isolates from the same patients and the phylogenetic tree (converted to an SNP-scaled tree using pyjar) (13) as input parameters.

The DBGWAS pipeline (14) was employed to detect the genomic signatures exclusively shared among the current NBP isolates. Moreover, to assess associations between an input phenotype and genome structures, this pipeline generates "unitig" consisting of k-mers using GATB, (15) and also conducts bugwas (16) for significant associations between unitigs and a phenotype of interest. Thus, to compute a gene database of the 376 *M. massiliense* isolates, we first annotated each genome using dfast-core version 1.2.18. (17) The resulting gene annotations were used with the Roary software and the "-cd 100" option (18). As an objective variable of the phenotype, the

NBP isolates were set to 1 (n = 51), whereas other *M. massiliense* isolates were set to 0 (n = 325). We focused on unitigs with *P* values < 1×10^{-8} and their neighboring unitigs in the network graph outputs from DBGWAS. Orthologous genes between clinical isolates were plotted using genoPlotR (19).

Supplemental Figures

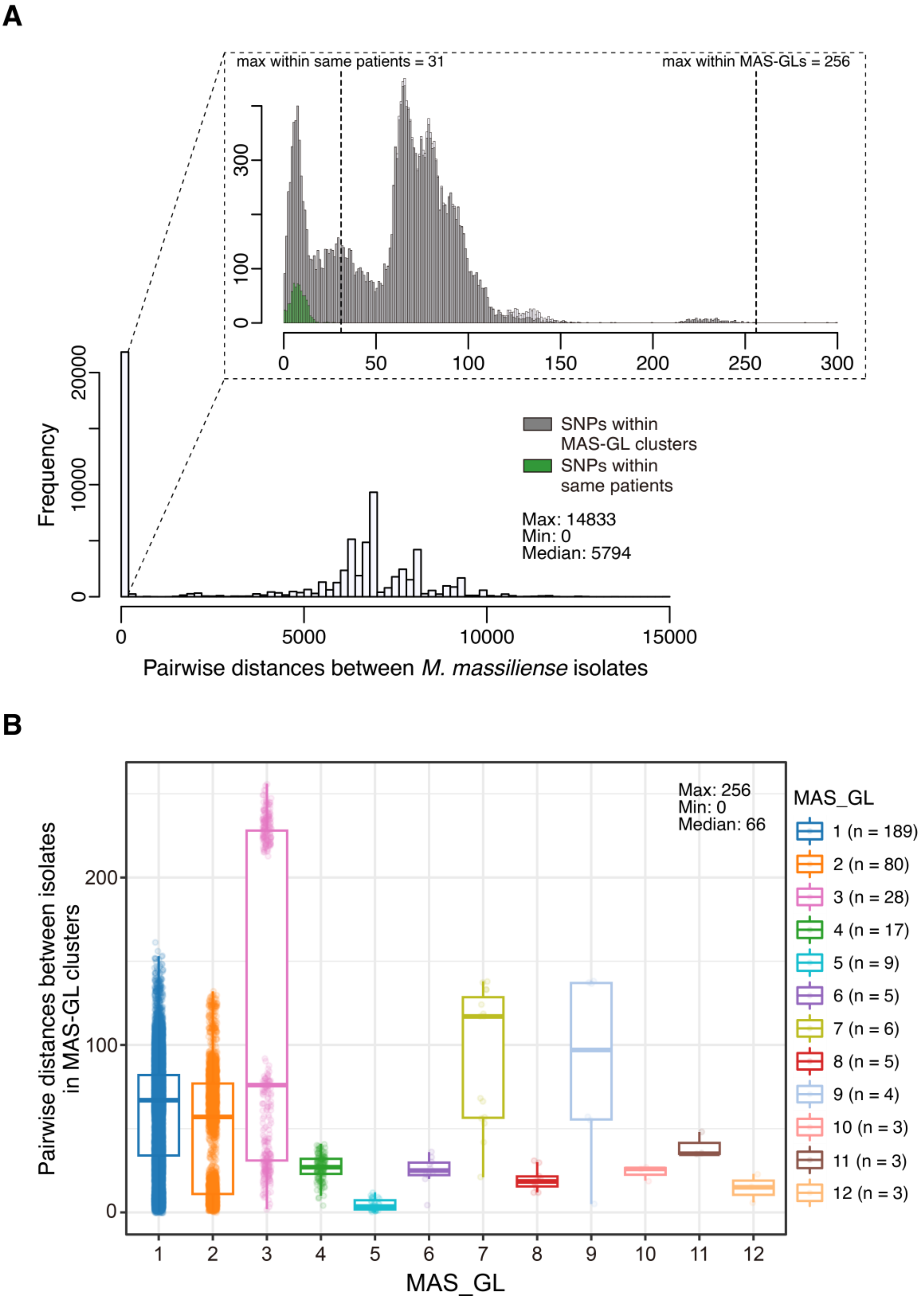

Supplementary Figure 1. Distribution of pairwise genetic distances between 384 *Mycobacterium abscessus* subsp. *massiliense* (*M. massiliense*) isolates from several countries.

(A) Histogram of pairwise single-nucleotide polymorphism (SNP) distances among global *M. massiliense* isolates. The SNP distances among isolates were calculated as described in Fig. 2 using snp-dists. The SNP distances among isolates within each of the MAS-GL clusters and consecutively obtained from the same patients are colored in gray and green, respectively. (B) Boxplot of pairwise SNP distances of isolates within each of 12 MAS-GL clusters.

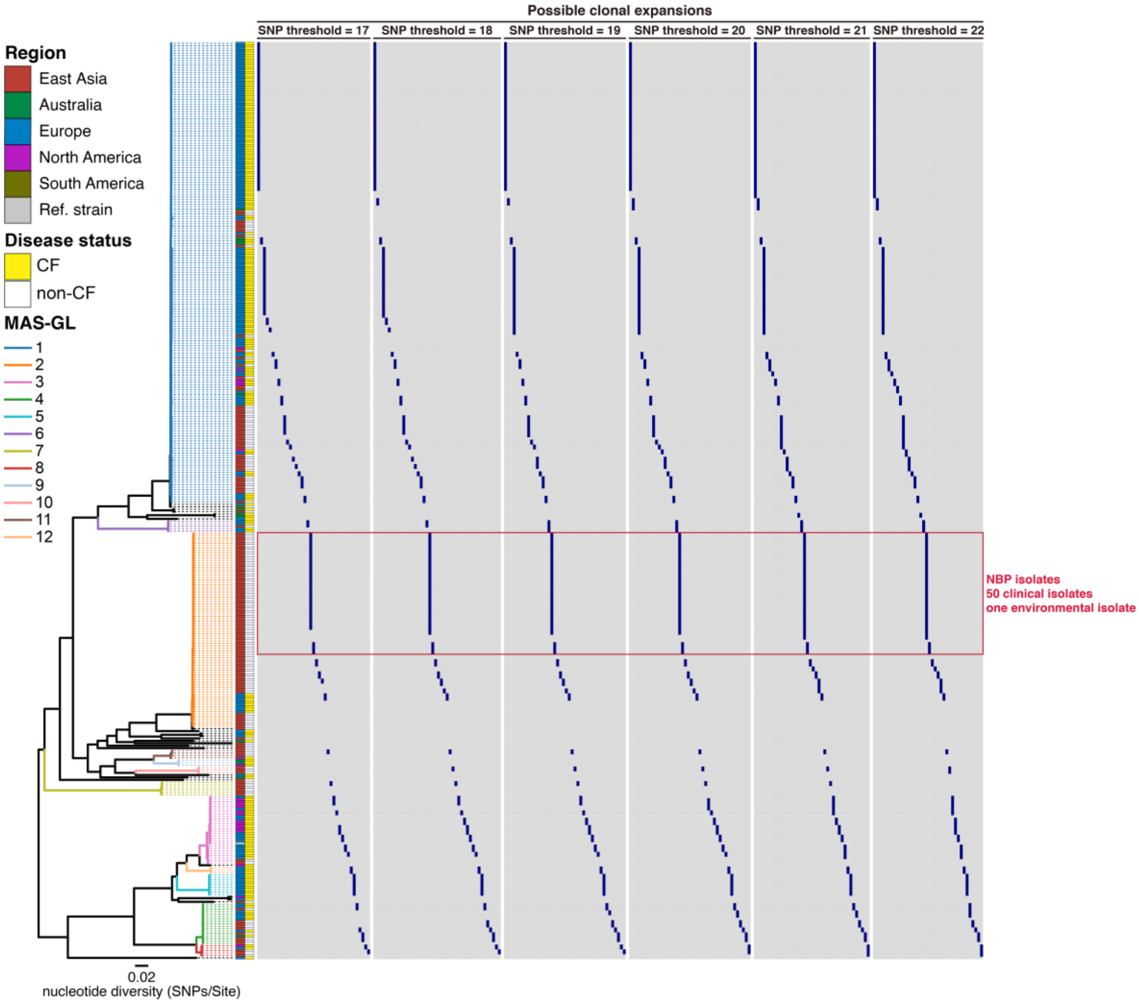

Supplementary Figure 2. Possible clonal expansions among *M. massiliense* isolates at several SNP thresholds.

The detection of possible clonal expansions (CEs) in the *M. massiliense* population was performed, as described in Fig. 3. This resulting tree and SNP thresholds (17–22 SNPs) were used as input for rPinecone. The blue boxes indicate the detected CEs. The indicated phylogenetic tree was estimated, as described in Fig. 1. The 12 MAS-GL clusters, the disease status (cystic fibrosis [CF] or non-CF) of the corresponding patients, and the region from which the clinical isolate was obtained are shown in Fig. 1.

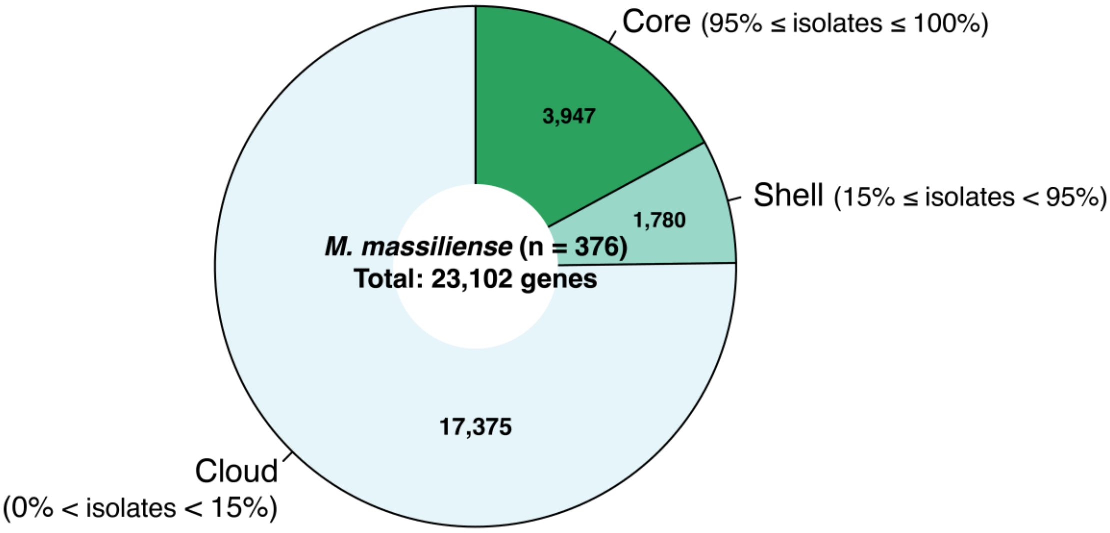

Supplementary Figure 3. Gene repertoire of 376 *Mycobacterium abscessus* subsp. *massiliense* (*M. massiliense*) isolates from several countries.

The pie chart presents the number of genes carried by *M. massiliense* among 376 strains from several countries. Genes from each isolate were annotated with DFAST-core (17), and the clustering of orthologous genes was performed using the Roary software (18). Genes that were carried by more than 95% of the isolates were designated as core genes, those carried by 15%–95% of the isolates were shell genes, and those carried by <15% of the isolates were cloud genes.

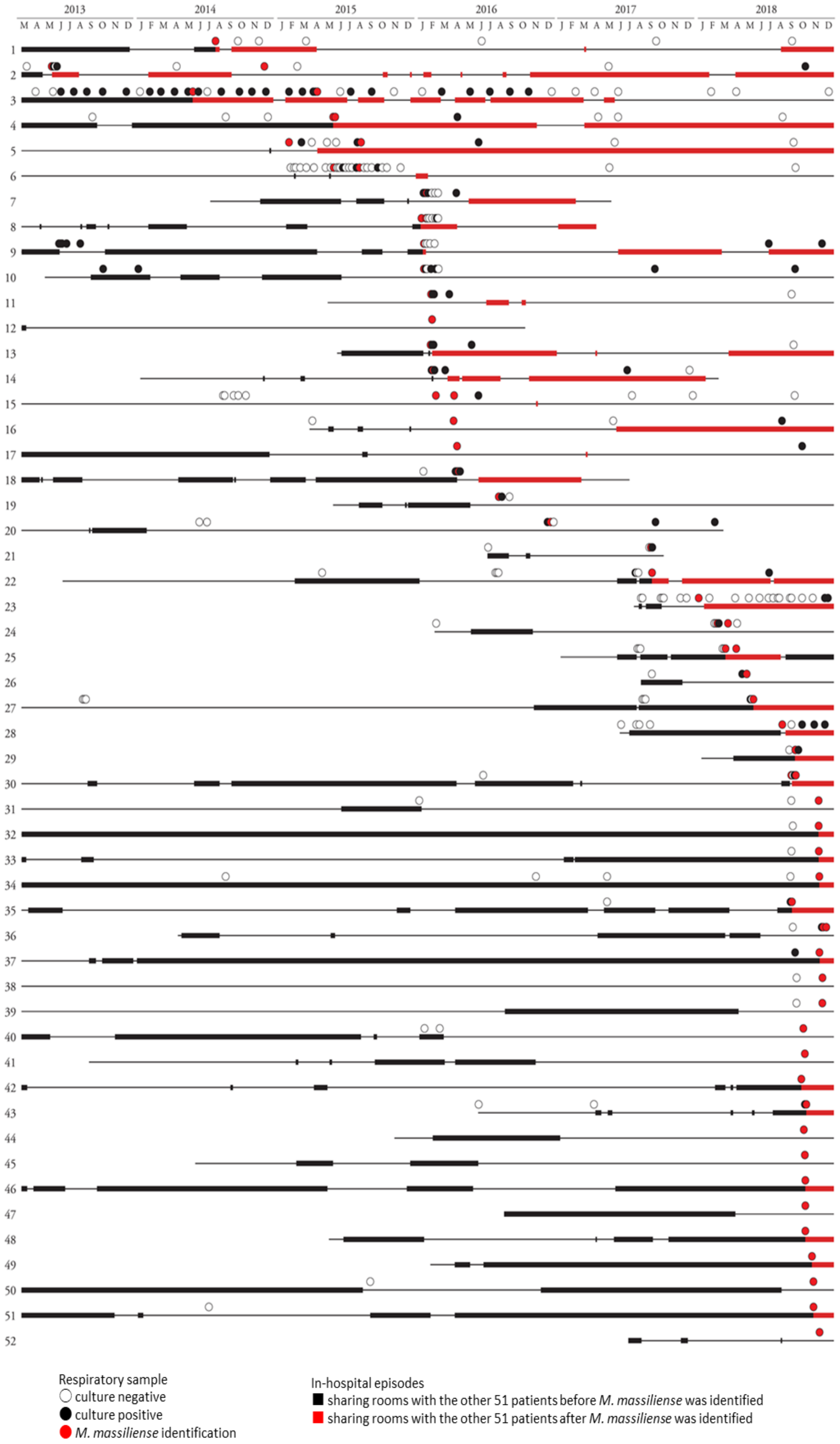
2013
2014
2015
2016
2017
2018
M A M J J A S O N D
J F M A M J J A S O N D
Respiratory sample
culture negative
culture positive
M. massiliense identification
In-hospital episodes
sharing rooms with the other 51 patients before M. massiliense was identified
sharing rooms with the other 51 patients after M. massiliense was identified

Supplementary Figure 4. Opportunities for patient-to-patient transmission within the hospital.

The timelines of individual patients with *M. massiliense* identification are shown. The transverse lines denote hospitalization at the Nishi–Beppu Hospital, and the vertical lines denote the duration of sharing rooms with other patients with *M. massiliense* isolation. The circles denote sputum samples (culture negative [white]; culture positive [black]). The timelines become red after a positive sputum sample. All patients had opportunities for transmission, whereas only one patient (#38).

Supplemental Tables

Supplementary Table 1. ***Mycobacterium abscessus*** **subsp.** ***massiliense*** **isolates from 52 patients listed by date and bed location**

Case	Date	Floor	Room	Case	Date	Floor	Room
1	Aug-1-14	1	117	27	May-16-18	2	213
2	Dec-8-14	1	114	28	Aug-15-18	5	506
3	Apr-17-15	1	112	29	Sep-4-18	5	507
4	May-29-15	2	201	30	Sep-5-18	1	106
5	Aug-6-15	1	104	31	Sep-6-18	5	501
6	Aug-12-15	2	206	32	Sep-10-18	1	102
7	Jan-26-16	1	103	33	Sep-10-18	1	101
8	Jan-26-16	1	102	34	Sep-10-18	1	101
9	Jan-26-16	1	114	35	Sep-13-18	1	106
10	Jan-27-16	2	217	36	Sep-18-18	2	205
11	Feb-8-16	5	520	37	Sep-20-18	2	201
12	Feb-8-16	5	504	38	Sep-25-18	3	302
13	Feb-8-16	5	514	39	Sep-26-18	4	401
14	Feb-9-16	5	507	40	Oct-4-18	2	206
15	Feb-25-16	2	203	41	Oct-4-18	2	208
16	Apr-7-16	1	112	42	Oct-10-18	1	113
17	Apr-12-16	1	116	43	Oct-16-18	1	115
18	Apr-18-16	1	106	44	Oct-16-18	3	303
19	Aug-12-16	1	114	45	Oct-16-18	4	415
20	Dec-5-16	5	518	46	Oct-17-18	1	113
21	Sep-8-17	5	507	47	Oct-18-18	4	403
22	Sep-14-17	5	506	48	Oct-19-18	1	117
23	Jan-4-18	2	216	49	Nov-1-18	2	201
24	Feb-19-18	2	204	50	Nov-5-18	2	202
25	Mar-9-18	5	506	51	Nov-5-18	2	213
26	Apr-25-18	1	102	52	Nov-20-18	2	211

Cases were numbered chronologically in the order in which *M. massiliense* was isolated.

Supplementary Table 2. ***Mycobacterium abscessus*** **subsp.** ***massiliense*** **isolates from 52 patients listed by year and location**

	Year				
**Floor**	**2014**	**2015**	**2016**	**2017**	**2018**
E5			11, 12, 13, 14, 20	21, 22	25, 28, 29, 31
E4					39, 45, 47
E3					38, 44
E2		4, 6	10, 15		23, 24, 27, 36, 37, 40, 41, 49, 50, 51, 52
E1	1, 2	3, 5	7, 8, 9, 16, 17, 18, 19		26, 30, 32, 33, 34, 35, 42, 43, 46, 48

Cases are numbered chronologically based on the order of *M. massiliense* isolation. E1–E5 denote the first to fifth floors of the east ward, respectively.

Supplementary Table 3. Characteristics of 52 patients from whom *Mycobacterium abscessus* subsp. *massiliense* was isolated

Variables	Number (%) or median (IQR)
Total patients	52
Women	27 (52)
Age, years	64 (43–72)
BMI, kg/m^2	19.7 (17.3–23.2)
Tube feeding	50 (96)
Underlying diseases	
Chronic obstructive pulmonary disease	1 (2)
Heart failure	13 (25)
Cerebrovascular diseases	6 (12)
Chronic kidney disease	9 (17)
Malignancy	7 (13)
Connective tissue disease	2 (4)
Diabetes mellitus	10 (19)
Duration of hospital stay, days	3,084 (1,920–5,576)
Days from admission to first detection of *M. massiliense*	2,195 (748–3,924)
Smear positive	50 (96)
Reason for sputum examination	
Fever or respiratory symptoms	17 (33)
Abnormal chest radiological findings	14 (27)
Screening	28 (54)
Treatment for *M. massiliense* infection	0 (0)
Death	11 (21)
Cause of death	
Respiratory failure attributable to *M. massiliense* infection	0 (0)
Respiratory failure attributable to other infectious diseases	4 (8)
Sepsis from urinary tract infection	2 (4)
Cardiac failure	2 (4)
Malignancy	2 (4)
Acute renal failure	1 (2)

BMI, body mass index; IQR, interquartile range

Supplementary Table 4.

Case	Subspecies	Contigs	Total length	GC (%)	N50	Coverage	Genome fraction (%)[a]	Contamination[b]
1*	MAS[c]	54	7335663	57.4	418264	68.3	90.7	59.2
2	MAS	14	5208577	64.1	804672	90.3	90.8	0.2
3	MAS	13	5209011	64.1	805338	103.3	90.8	0.2
4	MAS	15	5033219	64.2	506736	114.3	89.9	0.2
5	MAS	15	5032630	64.2	805466	97.3	89.9	0.2
6	MAS	14	5006352	64.2	507402	123.6	89.5	0.2
7	MAS	11	5032590	64.2	804672	143.0	89.9	0.2
8	MAS	12	5033315	64.2	749483	162.2	89.9	0.2
9	MAS	17	5017667	64.2	417935	135.4	90.8	0.2
10	MAS	13	5032869	64.2	805338	101.6	89.9	0.2
11	MAS	14	5032944	64.2	677198	189.5	89.9	0.2
12	MAS	13	5033023	64.2	805466	96.8	89.9	0.2
13	MAS	12	5098604	64.2	506736	169.8	89.9	0.3
14	MAS	12	5033958	64.2	955119	172.6	89.9	0.2
15	MAS	16	5209867	64.1	506865	162.4	90.8	0.2
16	MAS	13	5033324	64.2	506864	123.3	89.9	0.2

17	MAS	10	5219391	64.1	1011249	138.8	90.8	0.2
18	MAS	14	5206663	64.1	1011249	99.0	90.7	0.2
19	MAS	11	5188605	64.1	529162	73.8	90.4	0.2
20	MAS	9	5032934	64.2	955119	96.5	89.9	0.2
21	MAS	20	5036758	64.2	416654	113.4	89.9	0.2
22	MAS	11	5032786	64.2	734458	116.9	89.9	0.2
23	MAS	13	5006386	64.2	506736	118.9	89.6	0.2
24	MAS	10	5032823	64.2	953815	99.9	89.9	0.2
25	MAS	10	5034863	64.2	955119	124.3	89.9	0.2
26	MAS	10	5032560	64.2	603927	124.2	89.9	0.2
27	MAS	10	5033120	64.2	678490	117.5	89.9	0.2
28*	MAS	77	7862522	62.5	603927	85.8	89.9	83.0
29	MAS	9	5032906	64.2	955119	114.0	89.9	0.2
30	MAS	9	5032927	64.2	955119	103.1	89.9	0.2
31	MAS	12	5033187	64.2	603927	111.4	89.9	0.2
32	MAS	10	5033157	64.2	625333	104.2	89.9	0.2
33	MAS	8	5015484	64.2	1021328	129.9	90.8	0.2
34	MAS	11	5033032	64.2	955467	111.2	89.9	0.1

35	MAS	10	5032792	64.2	955119	107.1	89.9	0.2
36	MAS	9	5032934	64.2	955119	115.6	89.9	0.2
37	MAS	10	5032926	64.2	603927	118.6	89.9	0.2
38	MAS	11	5033112	64.2	603927	117.3	89.9	0.2
39	MAS	18	5033968	64.2	439420	110.0	89.9	0.2
40	MAS	9	5032336	64.2	804672	110.0	89.9	0.2
41	MAS	9	5032473	64.2	955467	109.8	89.9	0.2
42	MAS	13	5208742	64.1	529162	120.2	90.8	0.2
43	MAS	9	5032951	64.2	804800	109.9	89.9	0.2
44	MAS	11	5033178	64.2	603927	112.6	89.9	0.2
45	MAS	9	5032934	64.2	955119	113.2	89.9	0.2
46	MAS	14	5056026	64.2	955119	112.3	90.3	0.2
47	MAS	9	5032846	64.2	955119	113.9	89.9	0.2
48	MAS	9	5098803	64.2	804800	111.2	89.9	0.3
49	MAS	10	5033328	64.2	955119	116.9	89.9	0.2
50	MAS	9	5032929	64.2	804800	110.1	89.9	0.2
51	MAS	13	5032891	64.2	804800	114.0	89.9	0.2
52	MAS	9	5032898	64.2	955119	96.9	89.9	0.2

E[d]	MAS	10	5033247	64.2	603927	91.0	89.9	0.2

[a] Genome fraction indicates percentage alignment to the reference genome *M. massilisense* JCM15300. [b] contamination from foreign sources was estimated by CheckM2. [c] *Mycobacterium abscessus* subsp. *massiliense* (identified by whole-genome sequencing data), [d] environmental isolate. [*] Clinical isolates from patients #1 and #28 were excluded from downstream genomic analyses because of the high proportion of non-*M. abscessus* reads.

Supplemental References

1. Bryant JM, Grogono DM, Greaves D, Foweraker J, Roddick I, Inns T, Reacher M, Haworth CS, Curran MD, Harris SR, Peacock SJ, Parkhill J, Floto RA. 2013. Whole-genome sequencing to identify transmission of Mycobacterium abscessus between patients with cystic fibrosis: a retrospective cohort study. Lancet 381:1551–1560.
2. Tettelin H, Davidson RM, Agrawal S, Aitken ML, Shallom S, Hasan NA, Strong M, de Moura VC, De Groote MA, Duarte RS, Hine E, Parankush S, Su Q, Daugherty SC, Fraser CM, Brown-Elliott BA, Wallace RJ, Jr., Holland SM, Sampaio EP, Olivier KN, Jackson M, Zelazny AM. 2014. High-level relatedness among Mycobacterium abscessus subsp. massiliense strains from widely separated outbreaks. Emerg Infect Dis 20:364–371.
3. Bryant JM, Grogono DM, Rodriguez-Rincon D, Everall I, Brown KP, Moreno P, Verma D, Hill E, Drijkoningen J, Gilligan P, Esther CR, Noone PG, Giddings O, Bell SC, Thomson R, Wainwright CE, Coulter C, Pandey S, Wood ME, Stockwell RE, Ramsay KA, Sherrard LJ, Kidd TJ, Jabbour N, Johnson GR, Knibbs LD, Morawska L, Sly PD, Jones A, Bilton D, Laurenson I, Ruddy M, Bourke S, Bowler IC, Chapman SJ, Clayton A, Cullen M, Daniels T, Dempsey O, Denton M, Desai M, Drew RJ, Edenborough F, Evans J, Folb J, Humphrey H, Isalska B, Jensen-Fangel S, Jönsson B, Jones AM, et al. 2016. Emergence and spread of a human-transmissible multidrug-resistant nontuberculous mycobacterium. Science 354:751–757.
4. Yoshida M, Chien JY, Morimoto K, Kinjo T, Aono A, Murase Y, Fujiwara K, Morishige Y, Nagano H, Jou R, Hasegawa N, Ato M, Hoshino Y, Hsueh PR, Mitarai S. 2022. Molecular epidemiological characteristics of mycobacterium abscessus complex derived from non-cystic fibrosis patients in Japan and Taiwan. Microbiol Spectr 10:e0057122.
5. Chklovski A, Parks DH, Woodcroft BJ, Tyson GW. 2022. CheckM2: a rapid, scalable and accurate tool for assessing microbial genome quality using machine learning. bioRxiv.
6. Fujiwara K, Yoshida M, Murase Y, Aono A, Furuuchi K, Tanaka Y, Ohta K, Ato M, Mitarai S, Morimoto K. 2022. Potential cross-transmission of mycobacterium abscessus among non-cystic fibrosis patients at a tertiary hospital in Japan. Microbiol Spectr 10:e0009722.
7. Kurtz S, Phillippy A, Delcher AL, Smoot M, Shumway M, Antonescu C, Salzberg SL. 2004. Versatile and open software for comparing large genomes. Genome Biol 5:R12.
8. Croucher NJ, Page AJ, Connor TR, Delaney AJ, Keane JA, Bentley SD, Parkhill J, Harris SR. 2015. Rapid phylogenetic analysis of large samples of recombinant bacterial whole genome sequences using Gubbins. Nucleic Acids Res 43:e15.
9. Stamatakis A. 2006. RAxML-VI-HPC: maximum likelihood-based phylogenetic analyses with thousands of taxa and mixed models. Bioinformatics 22:2688–2690.
10. Yu G, Smith DK, Zhu H, Guan Y, Lam TT. 2017. ggtree: an r package for visualization and annotation of phylogenetic trees with their covariates and other associated data. Methods Ecol

Evol 8:28–36.

11. Csardi G, Nepusz T. 2006. The igraph software package for complex network research. Inter J. Complex Syst.

12. Wailan AM, Coll F, Heinz E, Tonkin-Hill G, Corander J, Feasey NA, Thomson NR. 2019. rPinecone: Define sub-lineages of a clonal expansion via a phylogenetic tree. Microb Genom 5.

13. Pupko T, Pe'er I, Shamir R, Graur D. 2000. A fast algorithm for joint reconstruction of ancestral amino acid sequences. Mol Biol Evol 17:890–896.

14. Jaillard M, Lima L, Tournoud M, Mahé P, van Belkum A, Lacroix V, Jacob L. 2018. A fast and agnostic method for bacterial genome-wide association studies: Bridging the gap between k-mers and genetic events. PLoS Genet 14:e1007758.

15. Drezen E, Rizk G, Chikhi R, Deltel C, Lemaitre C, Peterlongo P, Lavenier D. 2014. GATB: Genome assembly & analysis tool box. Bioinformatics 30:2959–2961.

16. Earle SG, Wu CH, Charlesworth J, Stoesser N, Gordon NC, Walker TM, Spencer CCA, Iqbal Z, Clifton DA, Hopkins KL, Woodford N, Smith EG, Ismail N, Llewelyn MJ, Peto TE, Crook DW, McVean G, Walker AS, Wilson DJ. 2016. Identifying lineage effects when controlling for population structure improves power in bacterial association studies. Nat Microbiol 1:16041.

17. Tanizawa Y, Fujisawa T, Nakamura Y. 2018. DFAST: a flexible prokaryotic genome annotation pipeline for faster genome publication. Bioinformatics 34:1037–1039.

18. Page AJ, Cummins CA, Hunt M, Wong VK, Reuter S, Holden MT, Fookes M, Falush D, Keane JA, Parkhill J. 2015. Roary: rapid large-scale prokaryote pan genome analysis. Bioinformatics 31:3691–3693.

19. Guy L, Kultima JR, Andersson SG. 2010. genoPlotR: comparative gene and genome visualization in R. Bioinformatics 26:2334–2335.
